# Brain Changes Associated With Long-Term Ketamine Abuse, A Systematic Review

**DOI:** 10.3389/fnana.2022.795231

**Published:** 2022-03-18

**Authors:** Jurriaan F. M. Strous, Cees J. Weeland, Femke A. van der Draai, Joost G. Daams, Damiaan Denys, Anja Lok, Robert A. Schoevers, Martijn Figee

**Affiliations:** ^1^Department of Psychiatry, University Medical Center Groningen, Groningen, Netherlands; ^2^Amsterdam University Medical Center, Location Vrije Universiteit Amsterdam, Amsterdam, Netherlands; ^3^Arkin, Mental Health Service, Amsterdam, Netherlands; ^4^Amsterdam University Medical Center, Location Academic Medical Center, Amsterdam, Netherlands; ^5^Netherlands Institute for Neuroscience, Royal Academy of Arts and Sciences, Amsterdam, Netherlands; ^6^Icahn School of Medicine at Mount Sinai, New York, NY, United States

**Keywords:** ketamine, drug abuse, side effects, gray matter volume, white matter volume, connectivity

## Abstract

Recently, the abuse of ketamine has soared. Therefore, it is of great importance to study its potential risks. The effects of prolonged ketamine on the brain can be observationally studied in chronic recreational users. We performed a systematic review of studies reporting functional and structural brain changes after repeated ketamine abuse. We searched the following electronic databases: Medline, Embase and PsycINFO We screened 11,438 records and 16 met inclusion criteria, totaling 440 chronic recreational ketamine users (2–9.7 years; mean use 2.4 g/day), 259 drug-free controls and 44 poly-drug controls. Long-term recreational ketamine use was associated with lower gray matter volume and less white matter integrity, lower functional thalamocortical and corticocortical connectivity. The observed differences in both structural and functional neuroanatomy between ketamine users and controls may explain some of its long-term cognitive and psychiatric side effects, such as memory impairment and executive functioning. Given the effect that long-term ketamine exposure may yield, an effort should be made to curb its abuse.

## Introduction

Ketamine is an anesthetic agent acting as an uncompetitive antagonist at the N-Methyl-D-Aspartate (NMDA) receptor. More recently, ketamine has emerged as a promising antidepressant (Berman et al., 2000; Zarate et al., 2012; DeWilde et al., 2015; Albuquerque et al., 2016; Singh et al., 2016; Daly et al., 2018). In a meta-analysis including 201 patients with major depressive disorder (MDD), a single intravenous administration of ketamine was associated with a marked reduction in depression severity compared to placebo within 4 hours (Xu et al., 2016), and recently esketamine has been approved as a treatment for depression in both the United States and Europe (EMA, 2019; FDA, 2019).

Despite the promising short-term effects, ketamine recently has been emerging as a drug of abuse. The prevalence of ketamine abuse was 1.7% in the United Kingdom in 2008/2009 (4% lifetime) (Murphy and Roe, 2010; Zou and Tan, 2016), and around 1% in American college students (Maxwell, 2005). Chronic ketamine abuse in these countries was associated with long-term cognitive impairment, mood disorders, psychotic and dissociative symptoms, suggesting that prolonged ketamine use may indeed negatively affect brain structure and functioning. An alternative explanation is that primary depressive, psychotic or dissociative symptoms are reasons for ketamine self-medication rather than long-term side-effects. It should be noted that recreational dosages are much higher than clinical dosages, both per dose and cumulatively.

To date, the safety of prolonged ketamine administration has sparsely been investigated in humans in a prospective manner. The studies that have been done, have been conducted in clinical setting, with a much lower dose than the doses that are used recreationally. However, given the scarcity of research on the topic, these findings are worth mentioning. The most frequently reported side effects of short term ketamine (hours/days) are related to the nervous system, such as dissociation, sedation, headache, dizziness, blurred vision and memory impairment (Short et al., 2017). Small case series of ketamine administration in various doses for up to one year in patients with MDD or chronic pain suggest that some of these neural side effects may remain with prolonged ketamine use (Cvrcek, 2008; Szymkowicz et al., 2013). Contrastingly, another study suggests that prolonged add-on treatment with intranasal esketamine, which has recently been approved for treatment of treatment resistant depression (TRD) twice or thrice weekly did not worsen cognitive performance after 44 weeks of maintenance treatment compared to baseline in patients with treatment resistant depression (TRD) (EMA, 2019; FDA, 2019; Wajs et al., 2020).

Despite the absence of prospective studies, some first insights might be gained from retrospective cohort studies in recreational ketamine users (UNODC, 2016). We sought to systematically review all available studies measuring long-term structural and functional neuroanatomical differences between long-term recreational ketamine users and controls. The results of this review could provide some first insights into potential effects of prolonged recreational ketamine use which may inform clinicians better about the risks associated with ketamine abuse.

## Methods

### Search Strategy and Selection Criteria

Preferred Reporting Items for Systematic Reviews and Meta-analyses (PRISMA) guidelines were followed during the writing process (Moher et al., 2009). We formulated the following PICO: what neuroanatomical differences exist between long-term ketamine users compared to non-ketamine using controls. First, we performed a broad search to include all relevant studies investigating the effects of long-term ketamine use on all organ systems, for possible future reports. For this review, we focused on gray matter volume changes, loss of white matter integrity, differences in functional connectivity and activation patterns and receptor-binding after long-term recreational ketamine use in adults. The intervention of interest was repeatedly-dosed ketamine with a minimum duration of more than 14 days. The use of ketamine in recreational users was compared to non-drug-using controls or poly-drug users. Although we did not exclude studies in which subjects also used other drugs, we considered the limitation that differences other than ketamine use alone could exist between ketamine users and control subjects, including use of other drugs.

A scoping search identified key articles and search concepts. All key articles had to be retrieved by the systematic search strategy. The search concept combination can be displayed as ketamine AND (chronic OR long term OR abuse OR dependence OR known long term use effects OR induced adverse effects).

Medline, Embase and PsycINFO were systematically searched (JD JS, FD) from inception until February 2021 using the Ovid interface. The search strategy was designed by JD. A detailed search strategy can be obtained from the corresponding author.

After removal of duplicates, 11,438 records were retrieved. The titles and abstracts of these records were independently screened by authors JS, CW and FD based on the selection criteria. Afterwards, conflicting results between the screeners were resolved by consensus.

**Figure 1:**
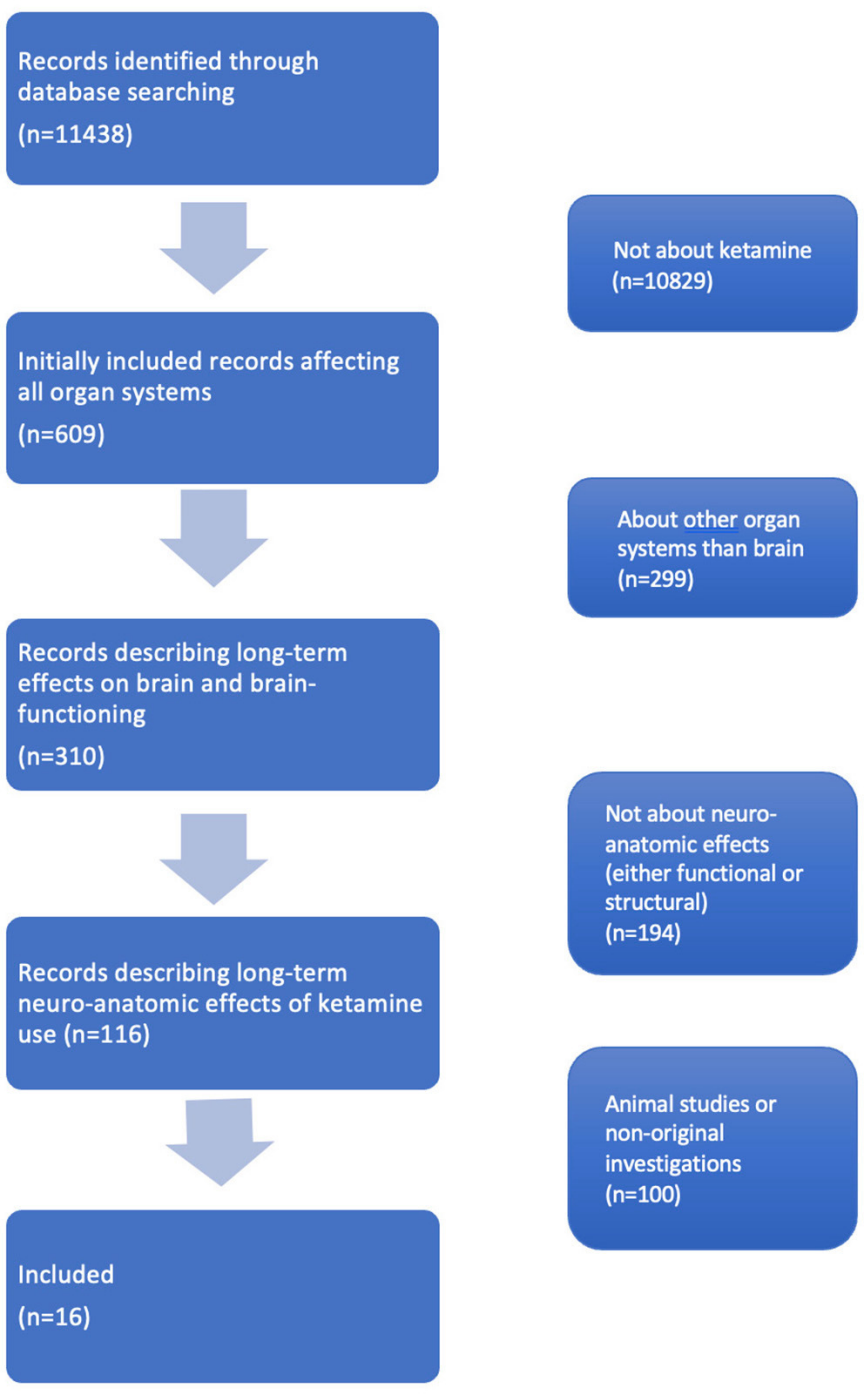
Inclusion flowchart.

Original studies about recreative ketamine use in which neuroanatomical measurements were performed, either structural or functional, were included. To obtain the articles meeting this inclusion criterion we first excluded all articles that were not about ketamine. Then we excluded all articles that were not about the brain. Subsequently we excluded articles that were only about brain function and not about neuro-anatomical outcomes (e.g., performance on cognitive tests). Lastly, we excluded papers that were about animals or were no original investigations. Then, we sorted the articles levels of evidence. Of all remaining articles, full-texts and one congress abstract were read. Finally, we found our complete dataset consisting of 16 studies (see Figure 1) for the inclusion flowchart.

### Data Extraction

Three of us (JS, FD, CW) independently extracted data from the retrieved articles: the N, characteristics of the ketamine consuming subjects, characteristics of the controls, significant differences between the ketamine consuming subjects compared to controls, the statistic measure and correlations (Tables 1–4). The quality of the studies was assessed using the Sackett Scale and the Oxford CEBM levels of evidence scale (Sackett, 1989; Howick et al., 2018).

**Table 1:** Structural differences: gray matter.

**References**	**N (ketamine vs. controls)**	**Ketamine subjects (ketamine use-other substance use-comorbid disorders)**	**Controls**	**Significant differences ketamine subjects compared to controls**	**Statistic measure**	**Correlation**
Liu et al. (2016)	181 (124–57)	25% mood disorder, 15.3% anxiety disorder. “Many” ketamine users used other drugs like cannabis or cocaine. Mean ketamine use: not mentioned Route of administration: not mentioned. Use of other drugs: not mentioned. Psychiatric and/or somatic illnesses: not mentioned. Male/female not mentioned Ketamine users performed worse than controls on intelligence and cognitive tasks. Presence of psychiatric or somatic disorder is not mentioned.	Healthy controls	Smaller gray matter volume in: -right orbitofrontal cortex (rOFC) -right medial prefrontal cortex (rMPFC) -left globus pallidus (lGP) -left hippocampus (lH) -right nucleus accumbens (rNAC) Higher gray matter volume in: -left caudate nucleus (lC)	Not reported	-Gray matter volumes in rOFC, rMPFC, rNAC were negatively correlated with ketamine dependence severity. -rOFC, rMPFC, lC, lGP, lH, rNAC volumes correlated with performance in cognitive tests.
Liao et al. (2011)	85 (41–44)	Mean age 26.9 years Male/female 33/8 Mean ketamine use: 2 g/day 3.4 years Ketamine was consumed by snorting Other drugs used in users: 100% Tobacco 73% Alcohol 68% Ecstasy 66% Amphetamine+ caffeine 56% Methamphetamine 20% Marijuana 15% Benzodiazepines 2,4% Heroin 0% Cocaine No history of neurologic disorder or psychiatric illnesses	Drug-free controls Mean age 26.3 years Male/female 34/10 0% Tobacco 20% Alcohol No history of neurologic disorder or psychiatric illnesses	Smaller gray matter volume in 1) left superior frontal cortex 2) right middle frontal cortex	Gray matter volume 1) Left superior frontal cortex: Voxel Z 4.48 2) Right middle frontal cortex voxel Z 4.81 P-cluster level < 0.05	Negative correlation with lower gray matter in left superior frontal cortex (*p =* 0.011) and right middle frontal cortex (*p =* 0.009) and duration of ketamine use (months). Negative correlation with lower left superior frontal cortex gray matter volume and estimated lifetime consumption (*p =* 0.022)
Chesters et al. (2016)	27 (14–13)	Age not mentioned Male/female not mentioned Mean ketamine use: not mentioned Other drugs: MDMA: 100% Cocaine: 100% Amphetamine: 93% Heroin: 50% Route of administration was not mentioned, age and gender were not mentioned. Other psychiatric or somatic disorders not mentioned	Polydrug controls Age not mentioned Male/female not mentioned Used drugs: MDMA: 46% Cocaine: 38% Amphetamine: 23% Heroin: 0% Other psychiatric or somatic disorders not mentioned	Smaller cortical matter volume and smaller cortical thickness in frontal lobe	Freesurfer analysis, *p =* 0.052, FDR corrected	
Wang et al. (2013)	21 (patient cohort)	Age: between 19 and 48 years Mean ketamine use: 1 g/day Route of administration was not mentioned Subjects were mostly below 30 years old, two individuals were above 30. Other drugs: One subject took a combination of ketamine, ecstasy and amphetamine. 1 subject was known to use amphetamine and ecstasy. No history of brain trauma or neurological disease. Psychiatric conditions were not mentioned	No controls	After ketamine addiction duration 1 yr: atrophy spots in superficial white matter of cortex 3 yrs: extension to internal capsule 4 yrs: atrophy spots in basal forebrain, cerebellum, pons and diencephalon; atrophy in small region in frontal, parietal and occipital cortex 5 yrs: atrophy of parahippocampal gyrus 6 yrs: atrophy lesions in corpus striatum. 7 yrs: extension of atrophy in frontal, parietal and occipital cortex. Lesions in midbrain. 10–12 yrs: all lesions as above.	Lesions were not quantifeid	In 1 subject, increased quantity (grams per year) ketamine use correlated with acceleration of cortical atrophy. In 1 subject, high amounts of polydrug (ecstasy, amphetamine, ketamine) correlated with acceleration of atrophy in basal prefrontal gyrus rectus
Hung et al. (2020a)	53 (34 users, 19 non-users)	Ketamine users were divided between adolescent (onset before age 20) onset users and adult onset users (onset after age 20).		1) Lower gray matter volume (GMV) in the left precuneus of ketamine users. The volume was	1) GMV 0.412 cm^3^ (adolescent), 0.48 cm^3^ (late onset) 0.51 cm^3^ (HC)	
		Age: 25.33 Mean ketamine use: not mentioned Male/female 26/8 Subjects testing positive on methamphetamine, opioids, ecstasy and marijuana were excluded. Participants had no major medical or neurological illness. The level of education of ketamine users was significantly lower than in the control group.	Age: 25.26 years Male/female 11/8 Participants had no major medical or neurological illness.	lower in the adolescent onset group than in the adult onset group. Lower GMV in the 2) right insula, 3) left inferior parietal lobule, 4) left DLPFC 5) lmOFC	*p* < 0.001 2) GMV 0.87 cm^3^ SD vs 1.11 cm^3^ 3) GMV 0.70 cm^3^ SD = 0.02 vs. 0.82 cm^3^ SD = 0.03 (*p* < 0.001) 4) 0.90 cm^3^ vs. 1.04 cm^3^ *p* < 0.001) 5) 0.63 cm^3^ vs. 0.73 cm^3^	

### Analysis

The results were subdivided into structural differences in gray and white matter, functional differences and effects on neurotransmission. Given the limited number of included studies and diversity of outcome measures in the studies, the data was deemed not suitable for meta-analysis. Therefore, we performed a conceptual synthesis of these heterogenous results. Because of these heterogenous results, we could not perform a quantitative bias analysis. Bias could play a role, since the 5 studies by Liao et al. (2010, 2011, 2012, 2016, 2018) were based on the same sample. For that reason, we compared the results if four of these five studies were left out of the analysis, to the situation in which all five studies were included.

## Results

We included 16 studies in our review, totaling 440 chronic ketamine users with a mean ketamine use of 2–9.7 years and 2.4 grams per day, compared to 259 drug-free controls and 44 poly-drug controls. Five studies were based on the same sample (Liao et al., 2010, 2011, 2012, 2016, 2018). The included studies described structural gray matter and white matter differences, differences in brain functionality and differences in neurotransmitter receptor binding. All retrieved studies were retrospective cohort studies, level IV on the Sackett scale or level 2b on the Oxford CEBM levels of evidence scale (Sackett, 1989; Howick et al., 2018).

### Structural Differences: Gray Matter

The results are shown in Table 1. A structural MRI study in 41 chronic ketamine users and 44 drug-free controls, found smaller gray matter volume in the left superior frontal cortex and the right middle frontal cortex in the ketamine group compared to controls (Liao et al., 2011). Subjects in this study used on average 2 grams of ketamine per day for a mean duration of 3.4 years from the start of ketamine use until the subject was included in the study. Subjects consumed the ketamine by snorting ketamine powder. The duration of ketamine use was negatively correlated with the gray matter volume in the left superior frontal gyrus (SFG) and right middle frontal gyrus (MFG) (Liao et al., 2011). Also, estimated total lifetime consumption of ketamine was negatively correlated with gray matter volume in the left SFG, but not in the right MFG (Liao et al., 2011). In a second structural MRI study, smaller cortical thickness in several regions in the right frontal area was observed in 14 chronic ketamine users compared to 13 poly-drug controls (Chesters et al., 2016). The route of ketamine administration was not mentioned. To assess the possible progression of brain changes following ketamine use over time, a third structural MRI study analyzed scans of 21 chronic ketamine users with varying durations of drug addiction, ranging from 0.5 to 12 years (Wang et al., 2013). Changes were observed in both white and gray matter across the internal capsule, basal forebrain, cerebellum and diencephalon. Longer use of ketamine was associated with more extensive cortical atrophy in the parahippocampal gyrus and frontal, parietal and occipital cortex. Interestingly, subjects that had been addicted to ketamine for 3 years or less showed less atrophy than subjects that were addicted more than 3 years. For example, the cortex was not affected in subjects with a ketamine addiction of < 3 years, and the limbic system was not affected in subjects with an addiction of < 4 years. However, cortical atrophy occurred earlier than 3 years in a patient who had been using a high dose of 3 grams ketamine per day (Wang et al., 2013).

A structural MRI study with 124 ketamine chronic ketamine users (dose and duration of abuse not reported) found lower gray matter volume in the right orbitofrontal cortex (rOFC), the right medial prefrontal cortex (rmPFC), the left globus pallidus (lGP), the left hippocampus (lH) and the right nucleus accumbens (rNAC) in the ketamine group compared to 57 controls. Gray matter volumes in rOFC, rMPFC and rNAC were negatively correlated with ketamine dependence severity and gray matter volumes of the rOFC, rmPF, lCN, lGP, lH, and rNAC negatively correlated with cognitive performance (Liu et al., 2016; Tang et al., 2016). Different from other studies, this study also found higher gray matter volume in ketamine users compared to controls, i.e., in the left caudate nucleus.

A study with 34 chronic ketamine users and 19 healthy controls found lower gray matter volume in the right insula, the left dorsolateral prefrontal cortex (DLPFC), the rOFC and the left inferior parietal cortex in ketamine users compared to controls (Hung et al., 2020a). Within the ketamine users group, adolescent onset users were compared to adult-onset users. Adolescent-onset users showed a significantly smaller left precuneus volume than the adult-onset group and the healthy control group.

### Structural Differences: White Matter

The results are shown in Table 2. Using diffusion-weighted MRI scans, fractional anisotropy (FA) can be used for estimating white matter fiber density, myelination and axonal diameter. FA reductions were found in bilateral frontal and left temporoparietal white matter in 41 ketamine users with a mean use of 2 grams/day for 3.4 years, in comparison with 44 drug-free controls (Liao et al., 2010). FA in the left and right frontal white matter was negatively correlated with the total lifetime consumption of ketamine. Diffusivity can be divided in axial and radial diffusivity. Axial diffusivity is thought to be a measure of axonal density and radial diffusivity is thought to be related to the degree of myelination (Liao et al., 2010). In 16 ketamine users averaging 2.4 grams/day for 7.3 years, a lower level of axial diffusivity was found compared to 16 polydrug controls, especially in the frontal part of the right hemisphere (Edward Roberts et al., 2014). Axial diffusivity was significantly lower in eight white matter clusters in the right hemisphere in the ketamine group compared to the control group, the three largest being located in the frontal cortex (Edward Roberts et al., 2014). Also, probabilistic tractography was performed to investigate cortico-subcortical white matter connectivity profiles, which revealed that white matter connectivity between the caudate nucleus and the lateral prefrontal cortex was positively correlated with severity of long-term dissociative symptoms (Edward Roberts et al., 2014).

**Table 2:** Structural differences: white matter.

**References**	**N (ketamine vs. control)**	**Ketamine subjects (ketamine use-other substance use-comorbid disorders)**	**Controls**	**Significant differences ketamine subjects compared to controls**	**Statistic measure**	**Correlation**
Liao et al. (2010)	85 (41–44)	Mean age 26.9 years Male/female 33/8 Mean ketamine use: 2 g/day 3.4 years Ketamine was consumed by snorting 73% Alcohol 68% Ecstasy 66% Amphetamine + caffeine 56% Methamphetamine 20% Marijuana 15% Benzodiazepine 2,4% Heroin 0% Cocaine No history of neurologic disorder or other psychiatric illnesses Excluded if other substance dependence (excl. nicotine)	Drug-free controls Male/female 34/10 Mean age 26.3 years 20% Alcohol	Lower FA in 1) left frontal cortex 2) right frontal cortex 3) left temporoparietal cortex white matter.	1) z= 4.21 (*p =* 0.014) 2) z=3.96 (*p =* 0.014) 3) z=3.85 (*p =* 0.014)	Negative correlation between FA in left (*p =* 0.015) and right (*p =* 0.003) frontal white matter and total lifetime consumption(g)
Edward Roberts et al. (2014)	32 (16–16)	Mean age 27.0 years Male/female 9/7 Mean ketamine use: 16.7 g/week 7.3 years Route of administration was not mentioned Regular use: 56% Alcohol 0% MDMA 44% Cannabis 19% Benzodiazepines 38% Cocaine Presence of psychiatric or somatic disorder is not mentioned.	Polydrug controls Mean age 28.5 years Male/female 11/5 Regular use: 75% Alcohol 0% MDMA 50% Cannabis 6% Benzodiazepines 44% Cocaine Presence of psychiatric or somatic disorder is not mentioned.	Less axial diffusivity in Eight right hemisphere clusters 1) anterior corona radiata and inferior fronto-occipital fasciculus (IFOF) 2) forceps minor 3) anterior forceps minor *Adjacent to parietal cortex:* 4) close to the intraparietal sulcus 5) close to posterior thalamic radiation and IFOF *Near SLF* 6) and somatosensory cortex 7) and corticospinal tract *Other areas* 8) sagittal stratum, inferior longitudinal fasciculus (IFL) and IFOF	1) T=3.972 671 voxels 2) T=3.966 177 voxels 3) T=4.345 34 voxels 4) T=4.71 173 voxels 5) T=3.482 165 voxels 6) T=3.256 22 voxels 7) T=3.637 92 voxels 8) T=2.49 38 voxels	
Liang et al. (2020)	180 60 (ketamine users) 64 (ketamine + polysubstance users) 56 (non-users)	*Primarily Ketamine Users* Mean age: 26.0 years Male/female 37/23 Ketamine Use 3.2 g/day 76 months Regular use: Cocaine 29.3% Metamphetamine 16.9% Marijuana 10.2% Hallicunogens 31.6% Presence of psychiatric or somatic disorder is not mentioned *Ketamine + Polysubstance users* Mean age 25.6 Male/female 36/28 Mean ketamine use: 3 g/day 77.7 months Regular use: Cocaine 69.2% Methamphetamine 29.7% Marijuana 27.7% Hallicunogens 27.7% Presence of psychiatric or somatic disorder was not mentioned.	*Non-users* Mean age 23.9 Male/female 24/32 No drug use is reported. Presence of psychiatric or somatic disorder was not mentioned.	1) Both primarily ketamine users and ketamine + polysubstance users had larger caudate nuclei than the non-drug controls. 2) Both ketamine groups had larger white matter volumes throughout the whole brain. 3) The K+polysubstance abuse showed even higher white matter volumes.	White matter volume was measured as a percentage of total intracranial volume. 1) (*p =* 0.030) 2) (*p =* 0.046) 3) (*p =* 0.011 compared to controls)	Earlier age of ketamine (both the primarily K and K+polysubstance users) use predicted larger white matter volumes.

*FA, fractional anisotropy; IF, inferior longitudinal fasciculus; IFOF, inferior fronto-occipital fasciculus; SLF, superior longitudinal fasciculus*.

In a study by Liang et al. (2020), ketamine users had larger caudate volume and total white matter volume than non-drugs controls. Ketamine users that also used stimulants had even larger white matter structures, suggesting an additive effect of ketamine and these stimulants. Participants in this study were asked to refrain from drug use 4–7 days before imaging was performed (Liang et al., 2020). The authors of this study hypothesize that the higher gray and white matter volumes reported in this study in ketamine users may not necessarily reflect neurotoxicity but rather represent positive compensatory changes as they were associated with less long-term cognitive impairments and depression. Opposingly, one could hypothesize that increased white matter volume may imply that an underlying low grade inflammatory edematous process exists, comparable to edema observed after brain injury (Weimer et al., 2017).

### Functional Differences

The results are shown in Table 3. Using resting-state functional MRI (fMRI), Liao al. investigated the functional connectivity between the thalamus and specific cortical regions in 40 chronic ketamine users with a mean use of 2 grams/day for 3.4 years, compared to 88 drug-free controls (Liao et al., 2016). Lower functional connectivity was found between the thalamus and the motor-, posterior parietal- and prefrontal cortex. Functional connectivity between the posterior parietal cortex and right lateral dorsal nucleus was significantly correlated to individual ketamine craving scores (Liao et al., 2016). Resting-state fMRI can also be used to measure regional homogeneity (ReHo). ReHo describes the summarized local functional connectivity between a voxel and its neighboring voxels. This is an index of network centrality, showing the importance of a voxel in a functional network. In 41 chronic ketamine users with a mean use of 2 grams/day for 3.4 years compared to 44 drug-free controls, lower ReHo in the right anterior cingulate cortex and higher ReHo in the left precentral frontal gyrus were found (Liao et al., 2012). The higher ReHo in the left precentral frontal gyrus was negatively correlated with estimated total lifetime ketamine consumption and ketamine craving (Liao et al., 2012). This may suggest that ReHo is initially increased more by ketamine use but that this increase eventually decreases with more prolonged and intensive use, which may alter functional organization in frontal networks. However, since subjects had to be abstinent from ketamine for only 48 hours, and since the direct effect of ketamine can last for more than 48 hours, the altered frontal network organization might also be a direct result of ketamine instead of a long-term side effect (Zarate et al., 2012). In the same sample, the authors compared smoking chronic ketamine users with non-ketamine smokers and with non-ketamine, non-smokers by performing fMRI. They found a higher activation in the anterior cingulate cortex (ACC) in response to ketamine cues. Also, ketamine subjects showed lower activation in the cerebellum and the middle temporal cortex in response to natural rewarding (sexual) cues (Liao et al., 2018).

**Table 3:** Functional differences.

**References**	**N (ketamine vs controls)**	**Ketamine subjects (ketamine use-other substance use-comorbid disorders)**	**Controls**	**Significant differences ketamine subjects compared to controls**	**Statistic measure**	**Correlation**
Liu et al. (2016)	124–57	25% mood disorder, 15.3% anxiety disorder. “Many” ketamine users used other drugs like cannabis or cocaine. Mean ketamine use: not mentioned Male/female not mentioned Ketamine users performed worse than controls on intelligence and cognitive tasks Presence of psychiatric or somatic disorder was not mentioned.	Healthy controls	-Less functional connectivity in orbital part of right inferior gyrus, left anterior cingulate and paracingulate gyri, right superior temporal gyrus and bilateral vermic lobule VI of cerebellum. -More functional connectivity left middle occipital gyrus.	No test statistic reported	
Liao et al. (2016)	130 (41–89) rsfMRI	Mean ketamine use: 2 g/day 3.4 years Mean age 26.8 years Male/female 33/7 Ketamine was consumed by snorting 100% Tobacco 75% Alcohol 70% Ecstasy 15% Diazepam 20% Marijuana 58% Methamphetamine 68% Amphetamine+caffeine No history of neurologic disorder or other psychiatric illnesses Excluded if other substance dependence (excl. nicotine)	Drug-free controls Mean age 27.1 years Male female 70/18 50% Tobacco 55% Alcohol No personal history of neurologic or psychiatric illnesses	Less thalamocortical connectivity between ketamine users and healthy controls. *connectivity of thalamic nuclei with:* *prefrontal cortex* 1) left medial dorsal nucleus *motor/supplementary motor area* 2) left ventral posterior lateral nucleus 3) right ventral lateral nucleus *posterior parietal cortex* 4) right pulvinar nucleus 5) left pulvinar n. 6) left medial dorsal n. 7) right ventral lateral n. 8) right lateral dorsal n.	1) T=3.72, voxel 270 mm^3^ 2) T=3.41 v=459 3) T=3.34 v=1354 4) T=3.72 v=243 5) T=2.76 v=108 6) T=4.32 v=108 7) T=3.20 v=162 8) T=3.53 v=405	Less thalamocortical connectivity in posterior parietal cortex and individual craving scores. (*p* < 0.05) A correlation between total ketamine intake and craving scores. (*p =* 0.003)
Liao et al. (2012)	85 (41–44) rsfMRI	Mean ketamine use: 2 g/day 3.4 years Mean age 26.9 years Ketamine was consumed by snorting 0% Cocaine 2,4% Heroin 15% Diazepam 20% Marijuana 56% Methamphetamine 63% Amphetamine+caffeine No history of neurologic disorder or other psychiatric illnesses Excluded if other substance dependence (excl. nicotine)	Drug-free controls Mean age 26.3 No history of neurologic or psychiatric illnesses	1) Lower ReHo in right anterior cingulate cortex. 2) More ReHo in left precentral frontal gyrus.	1) peak T=4.32 *p* < 0.001 voxel Z = 4.09 2) peak T=4.85 *p* < 0.001 Z = 4.54	Higher ReHo in left precentral gyrus negatively correlated with estimated total lifetime consumption (*p =* 0.035) and ketamine craving (*p =* 0.007).
Li et al. (2017)	56 (36–20) rsfMRI	Mean ketamine use: 4.9 years (M & F, dose not recorded) Mean age 25.2 (M) 27.5 (F) Male/female 26/10 Route of administration was not mentioned Mean tobacco use: 8.4 years (M) 12.1 years (F) Mean alcohol use: 4.3 years (M) 6.7 years (F) Subjects had no major medical or neurological illness	Drug-free controls Mean age 25.3 (M) 25.1 (F) Male/female 11/9 Mean tobacco use: 2.5 years (M) 0 years (F) Mean alcohol use: 5.2 years (M) 1.9 years (F) Subjects had no major medical or neurological illness	1) No difference in sgACC connectivity 2) Positive connectivity between sgACC and rostral ACC/medial prefrontal cortex/medial orbitofrontal cortex, bilateral temporal poles/superior temporal gyri, bilateral caudate head/ventral striatum/anterior thalamus and negative connectivity to bilateral occipital cortex, bilateral inferior frontal cortex/anterior insula (rIFC/AI), bilateral middle frontal gyrus and left angular gyrus	1) A two sample *t*-test was performed 2) A one sample *t*-test was performed	- sgACC-OFC connectivity negatively correlated with CES-D (*p =* 0.00001, *r* =-0.66) in KU (M & F) - sgACC-STG connectivity positively correlated with CES-D both in KU (*p =* 0.00002, *r* = 0.74) and in HC (*p* = 0.009, *r* = 0.74), men only - sgACC-dmPFC connectivity positively correlated with the CES-D in KU (*p* = 0.00003, *r* = 0.97), not in HC (*p* = 0.69, *r* = −0.16), female only
Morgan et al. (2014)	26 (11–15) fMRI	Mean ketamine use: 1.5 g/day 9.7 years Mean age 28 years Male/female 8/3 Route of administration was not mentioned 100% Tobacco 82% Alcohol 36% Cocaine 18% Ecstasy 73% Cannabis 1/11 personal history of mental illnesses somatic illness is not mentioned	Polydrug controls Mean age 26.13 years Male/female 10/5 87% Tobacco 100% Alcohol 6.7% Cocaine 0% Ecstasy 27% Cannabis 1/15 personal history of mental illnesses somatic illness is not mentioned	1) Less activity of right hippocampus and 2) Left parahippocampal gyrus in spatial memory task. 3) Less left caudate activation during memory updating.	1) t=2.77 *p =* 0.03 controls>ketamine users 2) t=2.81 *p =* 0.03 3) t=2.6, *p =* 0.048	
Chan et al. (2012)	6 (3–3) fMRI	Mean ketamine use: 1–2 g/day 2 years Mean age not mentioned Male/female not mentioned Route of administration was not mentioned Presence of psychiatric or somatic disease not mentioned	Drug-free controls Mean age not mentioned Male/female not mentioned Presence of psychiatric or somatic disease not mentioned	Lower number of activated areas in cerebellum during simple motor activities. Consecutively, subject drank 200 ml of red wine and repeated the motor task.	In controls, 55.7% of cerebellar volume was activated vs. 27.7% in ketamine users, and 21.1% in ketamine users + red wine condition.	
Liao et al. (2018)	129 (40 ketamine smokers, 45 non-ketamine smokers, 44 non-ketamine –non-smokers.	Mean ketamine use: not mentioned Other substance; nicotine. Subjects were excluded if any other substance use disorder was present.	-Nicotine smoking controls, -non ketamine, non-smoking	more activation in anterior cingulate cortex in response to *ketamine cues* 1) left anterior cingulate cortex 2) precuneus 3) cingulate gyrus 4) left inferior parietal cortex 5) right posterior cingulate 6) left occipital cortex (lingual gyrus) 7) right parietal cortex (supramarginal gyrus) *Smoking cues* 8) right frontal cortex (precentral gyrus) *Sexual cues* 9) left cerebellum 10) middle temporal cortex *Ketamine cues minus smoking cue* 11) left inferior parietal cortex 12) posterior cingulate/precuneus 13) left middle temporal cortex lower activation in left cerebellum and middle temporal cortex in response to sexual cues	1) peak T=4.45, voxel *z* = 4.28 Voxel size=378 mm^3^ 2) peak T=4.19, voxel *z* = 4.05 Voxel size=361 mm^3^ 3) peak T=3.96, voxel z=3.84 Voxel size = 77 mm^3^ 4) peak T=3.76, voxel *z* = 3.66 Voxel size=53 mm^3^ 5) peak T=3.76 voxel *z* = 3.66 Voxel size=45 mm^3^ 6) peak T=3.63 voxel z = 3.53 Voxel size=56 mm^3^ 7) peak T=3.62 voxel *z* = 3.52 Voxel size=53 mm^3^ 8) peak T=4.20 voxel *z* = 4.06 Voxel size=33 mm^3^ 9) peak T=4.65, voxel z = 4.46 Voxel size=123 mm^3^ 10) peak T=4.33, voxel z = 4.17 Voxel size=80 mm^3^ 11) peak T=4.22, voxel z = 4.14 Voxel size=130 mm^3^ 12) peak T=3.74, voxel z = 3.68 Voxel size=81 mm^3^ 13) peak T=3.58, voxel z = 3.53 Voxel size = 361 mm^3^	
Hung et al. (2020a)	53 (34 ketamine users, 19 controls)	Ketamine users were divided between adolescent (onset before age 20) onset users and adult onset users (onset after age 20). Age: 25.33 years Mean ketamin use: not mentioned Male/female 26/8 Subjects testing positive on methamphetamine, Opioids, ecstasy and marijuana were excluded. Participants had no major medical or neurological illness. The level of education of ketamine users was significantly lower than in the control group.	Participants had no major medical or neurological illness. Age: 25.26 years Male/female 11/8	Both 1) adolescent onset ketamine users and 2) adult onset ketamine users had higher functional connectivity between the left and right precuneus than 3) controls	1) *Z* = 0.21 SD = 0.03 2) *Z* = 0.25 SD = 0.03 3) *Z* = −0.02 SD = 0.03 *P* <0.001	
Hung et al. (2020b)	56 (36 ketamine users, 20 healthy controls	Age 25.2 years (M) 27.5 years (F) Male/female 26/10 Mean ketamine use: dose not recorded 59.4 months (M) 59.0 months (F) None of the ketamine users had a major medical, neurological illness.	Age 25.3 years (M) 25.1 years (F) Male/female 11/9	Ketamine users showed higher connectivity between 1) caudate and the dorsal anterior cingulate cortex 2) pallidum and bilateral cerebellum	1) voxel z=4.24 2) voxel z=5.28	The connectivity between putamen and lOFC correlated with months of ketamine use and BIS impulsivity scores (mediation analyes *p =* 0.00007–0.007)

*rsfMRI, resting-state functional magnetic resonance imaging; ReHo, regional homogeneity; KU, ketamine user; M, male; F, female; CES-D, Center of Epidemiological Study-Depression score; sgACC, subgenual anterior cingulate cortex; OFC, orbitofrontal cortex; STG, superior temporal gyrus; dmPFC, dorsomedial prefrontal cortex; fMRI, functional magnetic resonance imaging*.

Li et al. (2017) assessed resting-state functional connectivity of the subgenual anterior cingulate cortex (sgACC) in relation to depression scores of 36 chronic ketamine users with an average ketamine use of 4.9 years (dose not reported) compared to 20 drug-free controls. Overall, no difference in sgACC connectivity was found between groups, but in ketamine users higher depression scores correlated with lower sgACC connectivity to the right lateral and bilateral medial OFC. Further analysis revealed functional connectivity changes, with male and female ketamine users showing higher sgACC connectivity than controls to the bilateral superior temporal gyrus or dorsomedial prefrontal cortex (dmPFC), respectively (Li et al., 2017). Also, they found a correlation between higher sgACC connectivity with the dmPFC and higher depression scores in women, but not in men. Although ketamine has strong short-term antidepressant effects, the current data would suggest that chronic ketamine use may actually induce depression *via* sex-specific dysregulation of brain networks for positive and negative emotions. It remains unclear whether the altered connectivity patterns found in this study could be a direct result of ketamine. Being under the influence of ketamine was not an exclusion criterion for participation in this study.

Morgan et al. (2014) used fMRI to compare brain activity of 11 ketamine users with a mean use of 1.5 grams/day for 9.7 years, to 15 polydrug controls during a spatial memory task. Ketamine users showed lower activity in the right hippocampus and left parahippocampal gyrus compared to controls. Left caudate activity was greater in polydrug controls. These findings suggest an impact of long-term ketamine abuse on spatial memory processes associated with impaired (para)hippocampal activation. Lastly, in a small fMRI study using a motor task in which subjects had to flex and extend their upper limbs, three long-term ketamine users with a mean use of 1–2 grams/day for 2 years demonstrated less cerebellar activity compared to 3 drug-free controls (Chan et al., 2012). Being under the influence of ketamine was no exclusion criterion.

Lin et al. used resting state fMRI to compare a group of chronic ketamine users, many of which also used other drugs like cannabis or cocaine, to healthy controls (Liu et al., 2016). They found lower functional connectivity of the default mode network in the orbital right inferior frontal gyrus, left anterior cingulate gyrus, paracingulate gyri, right superior temporal gyrus and bilateral vermic lobule VI of the cerebellum. In contrast, they found higher functional connectivity in the left middle occipital gyrus.

In a study by Hung et al. (2020a), chronic ketamine users compared to healthy controls showed higher functional connectivity between the left DLPFC and the right inferior frontal/superior temporal gyrus and the left OFC and the right insula/inferior temporal gyrus. Within the ketamine users group, adolescent onset users were compared to adult onset users. Both the adult and the adolescent groups had higher functional connectivity between the left and right precuneus (Hung et al., 2020a).

In a pilot that studied white matter connectivity, chronic ketamine users showed higher connectivity between caudate nuclei and the dorsal anterior cingulate cortex (dACC). Ketamine users also showed a higher connectivity between the pallidum and the bilateral cerebellum. Furthermore, in ketamine users, the putamen showed higher connectivity to the OFC, which correlated with duration of ketamine use. Also, the ventral striatum (VS) showed lower connectivity with the right superior temporal sulcus (STS) and the left superior frontal gyrus (SFG) which was mediated by higher scores on the Barratt Impulsiveness Scale (BIS-11) (Hung et al., 2020b).

### Dopamine D_1_ Receptors

The results are shown in Table 4. One study investigated how long-term ketamine use affected neurotransmitter systems (Narendran et al., 2005). Dopamine D_1_ binding potential was studied using positron emission tomography (PET) imaging after intravenously injecting the selective D_1_ receptor radio ligand [^11^C]NNC 112 in 14 ketamine users with a mean use of 0.75 gram/week for 4.1 years and 14 drug-free controls. D_1_ receptor availability was significantly upregulated in the dorsolateral prefrontal cortex of ketamine users compared to controls, which could result from increased receptor density or affinity. D_1_ binding potential correlated with the total amount of ketamine consumption (Narendran et al., 2005).

**Table 4:** D_1_ receptor.

**References**	**N (ketamine vs. controls)**	**Ketamine subjects (ketamine use- other substance use-comorbid disorders)**	**Controls**	**Significant differences ketamine subjects compared to controls**	**Statistic measure**	**Correlation**
Narendran et al. (2005)	28 (14–14) PET-scan with radio-active ligand.	Mean ketamine use: 0.75 g/week 4.1 years Mean age 25 years Male/female 10/4 Route of administration was not mentioned 71% smokers. Absence of DSM-IV axis I diagnosis other than ketamine or cannabis abuse or dependence.	Drug-free controls Mean Age 25 years Male/female 9/5 43% smokers No history of neurologic or psychiatric illnesses incl. substance abuse.	Higher binding potential for D_1_ receptor in dorsolateral prefrontal cortex.	[^11^C]NNC 112 binding potential ml/g	Positive correlation between higher D_1_ binding potential and amount of consumed ketamine (*p =* 0.005).

*PET, positron-emitted tomography; DSM-IV, Diagnostic and Statistical Manual of Mental Disorders, 4th Edition*.

## Discussion

We systematically reviewed structural and functional brain changes after prolonged recreational ketamine use. Long-term ketamine abusers compared to controls displayed: (1) lower gray matter volume or cortical thickness in primarily the frontal, parietal and occipital lobes (2) lower white matter integrity in frontal and temporoparietal lobes (3) lower functional thalamocortical and corticocortical connectivity and abnormal frontal network organization (4) lower activity of brain regions for spatial memory and motor execution (5) higher functional connectivity between the DLPFC and the OFC and larger total white matter volume (6) higher dopamine D_1_ binding potential in the dorsolateral prefrontal cortex.

Including all five studies by Liao et al. could be a confound. Therefore, we also analyzed the results after excluding four of these five studies. As a result of this, finding (2) would change to “lower white matter integrity in right frontal and temporoparietal lobes” and/or finding (3) would not stand, depending on which articles were left out.

Many of the observed changes were correlated with the amount and duration of ketamine consumption, suggesting a possible dose dependent effect of prolonged ketamine on brain structure and function. Although the identified lower gray and white matter volumes or integrity could suggest direct neurotoxic effects of ketamine, the observed higher structural and functional connectivity and dopamine binding may suggest indirect compensatory effects. Together, these findings suggest that long-term intensive ketamine use may affect the structure and function of cortical gray and white matter, especially in frontoparietal regions.

It must be noted that the reported changes were dependent on the dosage and duration of ketamine use which were substantially higher than for clinical use, so our findings cannot be translated to clinical ketamine use. All subjects in these studies used at least 0.2 grams of ketamine twice weekly, which is around two to four times the recommended clinical dosage (25–50 mg intravenously twice or thrice weekly, equivalent to 50–100 mg intranasal ketamine, the most common route of administration in recreational use), with most subjects even consuming more than 1 gram daily which is equivalent to 25–70 times the clinical dose (Berman et al., 2000; Xu et al., 2016; Sanacora et al., 2017).

Nevertheless, this review does suggest that prolonged high dose ketamine use may have the potential to alter brain structure and function. Mechanistic support for such differences observed in long-term high dose ketamine comes from studies suggesting that exposure to ketamine induces apoptosis in adolescent primates and human brain cell cultures (Mak et al., 2010; Sun et al., 2014). In several of these preclinical studies chronic dosing regimens have been comparable to the regimens of chronic ketamine abusers. For example, 6 months of daily 60 mg intraperitoneal ketamine in mice was associated with reduced expression of GluA1-containg AMPA receptors and memory impairments (Ding et al., 2016) and 28 days of daily 30 mg/kg intraperitoneal ketamine was associated with reduced expression of glutamatergic receptor units GluA1, GluA2, GluN2A, and GluN2B, as well as a lower expression of synaptic proteins Syn and PSD-95, deteriorated cognitive skills, lower spine density, impairments in long-term potentiation and hampered transmission in the hippocampal CA1 area (Luo et al., 2020). Accordingly, in another study, the amount of parvalbumine positive interneurons in the CA1 region of mice hippocampi was reduced after 1 month of subcutaneous 16 mg/kg ketamine injections (Koh et al., 2016). These mice showed memory impairments, which is in line with a study in which parvalbumine positive interneurons are shown to play a role in hippocampal memory consolidation (Ognjanovski et al., 2017). Brains of mice and cynomolgus monkeys treated with 3 months of daily 30 mg/kg intraperitoneal ketamine (mice) or 1 mg/kg intravenous ketamine (monkeys) showed hyperphosphorylation of the tau protein, which could be interpreted as a neurotoxic effect since phosphorylation of tau protein is perceived as an aging marker (Yeung et al., 2010). Mice treated with 3 or 6 months daily subanesthetic doses (30 or 60 mg/kg) of intraperitoneal ketamine showed hyperphosphorylation of tau-protein hampering trafficking of AMPA-receptors, which in turn worsened signal transduction (Li et al., 2019). Another study suggested that impaired working memory after chronic exposure to ketamine in mice was mediated by upregulation of Gaba-5 subunit of the GABA_A_ receptor in the prefrontal cortex (Tan et al., 2011). The same group found that a 3-month ketamine treatment in mice was associated with upregulation of tyrosine hydroxylase (TH), the rate limiting enzyme in catecholamine-synthesis. The upregulation of TH might in turn be caused by upregulation of BDNF, which was also found after long-term ketamine use.

In conclusion, these animal studies may provide important clues for the potential neurotoxic effects of prolonged ketamine use. Prolonged ketamine may either up- or downregulate important regulatory neuronal proteins, potentially resulting in impaired neuronal functioning and cognitive performance.

A possible mechanism for the white matter changes identified in the reviewed recreational ketamine studies could be AMPA-receptor mediated excitotoxicity. In rats, ketamine was found to acutely elevate presynaptic glutamate in the prefrontal cortex at AMPA/kainite receptors (Moghaddam et al., 1997), and prolonged ketamine exposure may provoke cell death by regional glutamate-induced excitotoxicity. Excitation of AMPA receptors specifically induces axonal damage (Fowler et al., 2003), which could provide a potential mechanism for the prominent white matter changes observed after sustained ketamine exposure in three of the reviewed studies. Also, white matter changes in one of these studies preceded more widespread cortical atrophy with longer ketamine use, supporting that axonal cells are most vulnerable for glutamate-induced excitotoxicity by ketamine. However, these observations are still based on comparison between subjects rather than longitudinal data.

Our reviewed ketamine-associated brain changes might also explain some of the cognitive impairments and psychiatric symptoms observed in chronic ketamine users (Morgan et al., 2009, 2014). Impairments of working-, semantic-, spatial- and episodic memory in chronic ketamine users (Morgan and Curran, 2006; Chan et al., 2013) may be related to the observed atrophy and impaired function of brain regions underlying these memory functions, including the hippocampal complex, prefrontal and temporoparietal cortex. Findings of impaired executive functioning in chronic ketamine users (Narendran et al., 2005; Morgan et al., 2009) align with the reviewed frontostriatal impairments.

It needs to be considered however, that there may be a U-shaped dose-effect relation between ketamine and cognitive changes. In rats, different 5–7-days dosing regimens of ketamine yielded opposite effects on cognitive tasks in which the rats had to detect novel objects, or novel placement of objects. Whereas, low ketamine enhanced novelty detection compared to controls, higher doses impaired novelty detection (Schumacher et al., 2016).

The observed upregulation of dorsolateral prefrontal D_1_ receptors in ketamine users might be a compensatory mechanism for deficient prefrontal dopamine function underlying impaired working memory function (Narendran et al., 2005). Consistent with this, monkeys treated with another non-competitive NMDA antagonist, MK-801, showed lower performance on working memory tasks and lower prefrontal dopamine levels (Kakiuchi et al., 2001). Regarding psychotic symptoms, the observation of higher D_1_ binding potential in the dorsal prefrontal cortex after chronic ketamine use, may mirror similar findings in schizophrenia, as a compensation mechanism for prefrontal dopamine dysfunction and potentially explaining the high incidence of psychosis in chronic ketamine users (Abi-Dargham et al., 2002; Narendran et al., 2005; Uhlhaas et al., 2007; Morgan et al., 2009, 2010), although it might also be an epiphenomenon of an inflammatory pathway induced by ketamine (Wersinger et al., 2004; Lud Cadet et al., 2010). In addition, the observed hippocampal impairments in ketamine users may also have contributed to psychotic symptoms (Morgan et al., 2014). Altered hippocampal functioning is associated with the transition from a prodromal psychotic state to acute psychosis and repeated ketamine administration was able to induce these hippocampal impairments during progression to psychosis in a mouse model (Schobel et al., 2013). The dissociative symptoms after chronic ketamine use have been associated with differences in corticostriatal connectivity (Edward Roberts et al., 2014). Finally, despite the short-term antidepressant effects of ketamine, chronic ketamine use is associated with depressive symptoms (Morgan et al., 2009; Li et al., 2017), which correlates with altered functional connectivity between sgACC, OFC and ventromedial prefrontal cortex (Li et al., 2017), which are all areas involved in emotion regulation (Laitinen and Vilkki, 1973; Meyer et al., 1973; Talairach et al., 1973).

Caution is required in deeming prolonged ketamine use causal to the observed brain differences for several reasons. The reviewed brain differences might have been pre-existing and may have predisposed subjects to ketamine dependence. This is plausible considering that many of the observed brain differences concerned prefrontal regions that are crucial for inhibiting addictive behaviors. On the other hand, prefrontal gray matter reductions may have been initiated by ketamine use, further impairing inhibition and facilitating ketamine dependence. Similar mechanisms have been demonstrated for prefrontal changes associated with other types of drug abuse, including MDMA and cocaine (Cowan et al., 2003; Lim et al., 2008). Another finding that could explain transition to ketamine addiction in recreational users is lower thalamocortical connectivity, which may impair control over cognitive and emotional processes involved in drug-seeking behavior (Balleine et al., 2015). Although only a subset of recreational ketamine users are reported to develop dependence (Muetzelfeldt et al., 2008) and tolerance (Bonnet, 2015), the current review suggests several potential mechanisms for addiction that should be further explored to gain an understand of ketamine abuse.

Even though this review shows associations between long-term ketamine and structural and functional neuroanatomical differences, it holds important limitations. The results have been obtained from recreational ketamine users for whom we do not precisely know what dose of ketamine they used, which type of ketamine (racemic or esketamine) and whether they consumed pure ketamine or ketamine contaminated with other substances. This review shows that several functional and structural changes appear to correlate with duration and dose of ketamine consumption and are most striking after more than 3 years of high doses. The results of this review are not translatable to clinical ketamine regimens, since therapeutic dosages may lead to significantly less brain changes than recreationally dosed ketamine, or that it may take much longer to develop brain changes with therapeutic regimens. However, studies on long-term brain effects of therapeutic ketamine are lacking. Nonetheless, evidence exists for opposite clinical effects of low dose vs. high dose ketamine. Low dose, twice weekly ketamine schedules have strong antidepressant effects in depressed patients (Singh et al., 2016), whereas high dose daily schedules have been associated with depression in ketamine abusers (Morgan et al., 2009). Furthermore, low dose ketamine schedules in mice increased sprouting in the medial prefrontal cortex compared to saline injections (Li et al., 2010), whereas chronic self-administration of high dose ketamine in rats reduced glutamate receptor expression in the medial prefrontal cortex (Caffino et al., 2017). This may be in line with brain volume loss and reduced connectivity after high dosing recreational ketamine schedules in humans (Liao et al., 2010, 2011, 2012, 2016; Wang et al., 2013; Edward Roberts et al., 2014; Chesters et al., 2016), but enhanced brain connectivity after lower dose ketamine (Abdallah et al., 2017). Furthermore, long-term uncontrolled dosing may lead to ketamine-tolerance, as illustrated by a case report of a woman who became more depressed after administering herself increasing doses of ketamine (Bonnet, 2015), while short term controlled regimens in rats led to ketamine sensitization, as shown by increased locomotor activity after two ketamine injections compared to one injection (Rocha et al., 2017). Lastly, it should be noted that racemic ketamine and esketamine may have distinct toxicity profiles. Recreational users may mainly use racemic ketamine, whereas long-term treatment of depression mainly concerns esketamine which could be less toxic to the brain (Wajs et al., 2020).

A second limitation of the reviewed studies is that use of other substances including tobacco was more prevalent among ketamine users compared to the drug-free controls, although several studies included polydrug users as a control group. Therefore, the observed brain changes cannot indisputably be ascribed to ketamine alone. In addition, street ketamine might not be pure ketamine but could be contaminated with other drugs, which would strengthen this confounding. Also, ketamine abuse itself might give rise to abuse of other substances. For example, ketamine has an effect on mu-opioid receptors and its antidepressant effect might even be partially mediated by this receptor (Smith et al., 1980; Gupta et al., 2011; Williams et al., 2018). This effect on the mu-opioid receptor might give rise to opioid abuse. In one of the included samples, 50% of ketamine users had also been using heroin, which could have contributed to the observed brain changes (Sanacora and Schatzberg, 2015; Chesters et al., 2016). This interplay between ketamine and mu-opioid receptors might also apply to other neurotransmitter systems and associated drugs of abuse.

Third, since subjects were mostly recreational users, they might have used ketamine shortly before data were obtained. Therefore, the different functional connectivity patterns could in part be caused or influenced by the direct, short term effects of ketamine.

Fourth, most of the included subjects were of Asian ethnicity, which might have influenced outcomes for instance through genetic differences in drug metabolism. However, it has been shown that frequencies of cytochrome P450 variants responsible for ketamine metabolism do not vary significantly between people with Asian or Caucasian ancestry (Mizutani, 2003; Peltoniemi et al., 2016).

We consider a few methodological limitations as well. The included studies followed a cross-sectional and retrospective design with considerable variability among studies in terms of subject age, ketamine type and dosage. The data was insufficient to perform a meta-analysis. Additionally, five of the 16 studies were based on the same sample. It should be noted that in some studies, ketamine users had a mood disorder and for many of the studies it was unclear whether the ketamine users were diagnosed with another substance use disorder or another psychiatric illness. Part of the structural and functional neuroanatomical differences could therefore be attributed to these concomitant conditions. Finally, we were unable to receive a few records containing potentially relevant data.

In conclusion, prolonged high-dosed recreational ketamine use is associated with structural and functional brain differences. Why these differences exist, has not been established yet, but may follow patterns observed in animals, i.e., hyperphosphorylation of tau-protein and disrupting expression of several receptor subunits.

Despite its limitations, this review underscores the need for further study of potential risks associated with repeated administration of ketamine. To elucidate causal associations between brain changes and prolonged ketamine use, prospective and longitudinal imaging studies with controlled low-dose administrations are needed, ideally combined with cognitive tasks.

## Data Availability Statement

The original contributions presented in the study are included in the article/supplementary material, further inquiries can be directed to the corresponding author/s.

## Author Contributions

JS, CW, FD, and JD performed the literature search. JS, CW, and FD collected and interpreted the data. JS, CW, FD, JD, AL, DD, RS, and MF wrote this paper. All authors provided their consent for publication of this paper.

## Funding

This study was funded by a suicide prevention grant from ZonMw, the Netherlands Organization for Health Research and Development, Grant No. 537001004.

## Conflict of Interest

The authors declare that the research was conducted in the absence of any commercial or financial relationships that could be construed as a potential conflict of interest.

## Publisher's Note

All claims expressed in this article are solely those of the authors and do not necessarily represent those of their affiliated organizations, or those of the publisher, the editors and the reviewers. Any product that may be evaluated in this article, or claim that may be made by its manufacturer, is not guaranteed or endorsed by the publisher.
